# Pan-cancer analysis of the prognostic value of C12orf75 based on data mining

**DOI:** 10.18632/aging.203081

**Published:** 2021-06-01

**Authors:** Guangzhen Cai, Guannan Jin, Junnan Liang, Ganxun Li, Xiaoping Chen, Huifang Liang, Zeyang Ding

**Affiliations:** 1 Hepatic Surgery Center, Tongji Hospital, Tongji Medical College, Huazhong University of Science and Technology, Wuhan, Hubei, PR China; 2 Clinical Medicine Research Center for Hepatic Surgery of Hubei Province, Wuhan, Hubei, PR China; 3 Key Laboratory of Organ Transplantation, Ministry of Education and Ministry of Public Health, Wuhan, Hubei, PR China; 4 Department of Nephrology, Union Hospital, Tongji Medical College, Huazhong University of Science and Technology, Wuhan, Hubei, PR China

**Keywords:** chromosome 12 open reading frame 75 (*C12orf75*), pan-cancer analysis, prognostic biomarker, tumor microenvironment, carcinogenesis

## Abstract

The differential expression of chromosome 12 open reading frame 75 (*C12orf75*) is closely related with cancer progression. Here, we studied the expression levels of *C12orf75* and investigated its prognostic value in various cancers across distinct datasets including ONCOMINE, PrognoScan, GEPIA, and TCGA. The correlation between genetic alteration of C12orf75 and immune infiltration was investigated using the cBioPortal and TIMER databases. RNA interference was used to verify the influence of *C12orf75* knockdown on the biological phenotype of hepatocellular carcinoma cells. C12orf75 showed increased expression in most tested human cancers. The increased expression of *C12orf75* was related with a poor prognosis in urothelial bladder carcinoma and hepatocellular liver carcinoma, but it was surprisingly converse in renal papillary cell carcinoma. In urothelial bladder carcinoma and hepatocellular liver carcinoma, we observed positive correlations between the expression of *C12orf75* and the infiltration of immune cells, including B cells, CD8+ T cells, CD4+ T cells, macrophages, neutrophils, and dendritic cells. The knockdown of *C12orf75* in hepatocellular carcinoma cells suppressed the proliferation, migration, and invasion and arrested the cell cycle. This is the first report *C12orf75* has potential as a prognostic biomarker and therapeutic target for molecularly targeted drugs in urothelial bladder carcinoma, hepatocellular liver carcinoma, and renal papillary cell carcinoma.

## INTRODUCTION

Tumors remain a leading cause of morbidity and mortality worldwide. Poor prognosis related with high recurrence and progression rates remains the main challenge of cancer therapy, which is often complicated by an insidious onset, invasiveness, rapid growth, and metastasis [1]. In 2019, more than 1.5 million people were diagnosed with different types of cancer worldwide, with 600,000 patients in the United States alone [2]. The number of patients diagnosed with cancer is increasing, leading to a significant economic burden and health policy challenge. In 2018, bladder cancer was the ninth most common cancer [3], and hepatocellular carcinoma (HCC) was the fourth leading cause of cancer deaths in the world [4]. Even with the rapid development of tumor diagnosis and treatment methods in recent years, cancer remains a major threat to human health. Therefore, it is necessary to clarify the mechanisms of cancer occurrence and identify cancer-related biomarkers for prognosis and treatment.

Chromosome 12 open reading frame 75 (*C12orf75*) gene, located on 12q23.3, was first designated Overexpressed in Colorectal Carcinoma-1 (OCC-1). In the earliest research, the *C12orf75* gene was found to play a non-coding regulatory role, and it was confirmed to be expressed and up-regulated during the differentiation of mesenchymal stem cells into adipocytes [5, 6]. In recent years, *C12orf75* was found to have a close relationship with human cancers. In different colorectal cancer studies, *C12orf75* was found to either promote or inhibit cancer cell growth [7]. Similarly, *C12orf75* was reported to promote the apoptosis of breast cancer cells [8]. However, there was no research clarifying the association between *C12orf75* and other human cancer types in the previous literature. Based on the available studies, *C12orf75* appears to play dual roles, either promoting or inhibiting tumor progression depending on the cancer type, but there was a lack of broader studies in different human cancers.

To our best knowledge, this is the first study to comprehensively analyze *C12orf75* expression and its correlation with the prognosis of different types of malignant tumors using databases such as The Cancer Genome Atlas (TCGA), Oncomine, GEPIA, and PrognoScan. To reveal the possible mechanism of the dual roles of *C12orf75* in different cancers, the potential correlation between *C12orf75* expression and immune infiltration status was investigated using the TIMER and GEPIA databases. We conducted GO analysis and found pathways through which *C12orf75* possibly influences tumor biological phenotype in conjunction with the TCGA database. Finally, we verified the deduced potential function through experiments with cancer cell lines and analysis of patient samples.

## METHODS

### Difference expression analysis of *C12orf75*

The Oncomine database, the largest oncogene chip database, and integrated data-mining tool, was used to reveal the differentiation of *C12orf75* mRNA expression level between tumors and normal tissues in different types of carcinoma [9]. The Oncomine results were exhibited with the *p*-value of 0.001, the top 10% gene ranking, and fold change of 1.5. Meanwhile, Gene Expression Profiling Interactive Analysis 2 (GEPIA2) was used to demonstrate the expression differentiation of the *C12orf75* in different types of carcinoma and tissues by matching TCGA and GTEx data (normal tissue) [10]. The parameter settings were differential methods of ANOVA and log-scale of log2(TPM + 1).

### Data acquisition

We downloaded and processed TCGA mRNA expression data and phenotype data from the UCSC Xena browser(http://xena.ucsc.edu/) [11, 12]. For gene expression, the RNA-Seq (polyA+ Illumina HiSeq) data were downloaded as log2 (fragments per kilobase of transcript per million mapped reads upper quartile (FPKM)+ 1) values. All quantification files were selected, and all files in clinical sections were analyzed. From the differentially expressed gene found in TCGA, *C12orf75* was chosen for this study.

### Survival analysis

The R package *"limma"* was used to normalize the expression data of the *C12orf75* gene of TCGA-FPKM [13]. The median was as the cut-off value to divide the patients into high and low expression groups and then used the R package *"survival"* to analyze survival differences including overall survival (OS), disease-specific survival (DSS), disease-free interval (DFI), and progression-free interval (PFI) in different cancers between high and low groups. And Kaplan-Meier survival curves drawn based on differences of *C12orf75* expression were generated by the R package *"survival"* in different cancers. The *p*-values were calculated from the log-rank test and corrected using the Benjamini-Hochberg method. The *p*-values was considered as significant. PrognoScan (http://dna00.bio.kyutech.ac.jp/PrognoScan/index.html) was further applied in survival analysis around the association between prognostic and different expression levels of *C12orf75* expression in distinct carcinoma [14]. In all available microarray datasets of PrognoScan, the *C12orf75* expression level was searched to determine its prognosis relationship. R software package *"forestplot”* was used to imagine the survival analysis from PrognoScan [15]. And univariate and multivariate Cox survival analysis was also achieved by using the R package *"survival"* to verify the independence of *C12orf75* as a biomarker.

### Analysis of the clinicopathological features

HTSeq-fragments per kilobase of transcript per million mapped reads (FPKM) and the corresponding phenotype data were utilized to analyze the relationship between clinicopathological features and expression level of *C12orf75*. we used *"ggplot"* and *"ggpubr"* software packages in R to perform Plots and statistical analysis [16]. *p-*values were calculated using a non-paired Wilcox test.

### Genetic alteration analysis

The cBioPortal web (https://www.cbioportal.org/) was used to queries the genetic alteration features of *C12orf75* [17, 18]. The option of "Quick select" and "TCGA Pan-Cancer Atlas Studies" was selected. The alteration frequency, copy number alteration (CNA), and mutation type were depicted in the "Cancer Types Summary" module overall TCGA database.

### Immune infiltration analysis

The correlation between *C12orf75* expression and immune infiltration was discovered utilizing the TIMER (http://cistrome.org/TIMER/) and GEPIA databases [10, 19]. We analyzed the expression of *C12orf75* under the abundance of B cells, CD4+ T cells, CD8+ T cells, neutrophils, macrophages, and dendritic cells (DC). And the association between *C12orf75* expression difference and tumor purity was investigated. Besides the common investigation of immune cell types, investigating the relationship between *C12orf75* expression differences and individual markers of immune cells was utilized to distinguish possible subtypes of immune infiltration cells. The R&D Systems website (https://www.rndsystems.com/cn/resources/cell-markers/immune-cells) was applied to define gene markers in different types of immune cells. Similarly, in GEPIA, the correlation analysis between the expression of immune cell markers and *C12orf75* was carried out on the TCGA mRNA expression data set. The correlation coefficient was determined by the Spearman method and *p*-values were corrected using the Benjamini-Hochberg method.

### Methylation analysis

DNMIVD offered prognostic models based on DNA methylation and gene expression data from TCGA [20]. The association between the methylation of gene promoters and survival status was also accessible online.

### Enrichment analysis

The *"clusterProfiler"* R package was utilized to perform Gene Ontology (GO) enrichment analysis [21]. The data for biological process (BP), cellular component (CC), and molecular function (MF) were depicted as bubble charts. The R language software [R-3.6.0, 64-bit] (https://www.r-project.org/) was applied in this analysis. The enriched pathways were depicted with the "enrichplot" and *"ggplot2"* R packages at last.

### RNA interference

RNA interference was used to knock down *C12orf75*. Liver cancer cells were plated into each well of a 6-well plate. After 24 hours, in each well, those cells were transfected with 3.75 μl lipofectamine 3000 and 5 μl of siRNA oligo (20 μM) for 48 hours. Cells were re-digested and subjected to indicated assays. (*C12orf75* siRNA sequence: siRNA#1 GTGTCCAGTCAAACAAAGA; siRNA#2 CTACCATCTGAAGCTGTCA; siRNA#3 CTATGGAGGAGTATATGTT).

### RT-PCR

The *C12orf75* forward primer sequence was AGCCAAAGATGTAACAGAAGAATCCG, and the reverse primer sequence was ACAGCTTCAGATGGT AGGCCAAC. The Ct values of the indicated genes were normalized to those of the internal control GAPDH. Each experiment was repeated three times. The 2-ΔΔCt method was used to calculate relative expression.

### Cell viability assays

Under the manufacturer instructions, cell viability was examined utilizing the Cell Counting Kit-8 (CCK-8) (Beyotime Institute of Biotechnology, China). The indicated numbers of cells (counted using a Cellometer Mini, Nexcelom Bioscience, Massachusetts, USA) were plated into 96-well plates and cultured for 5 days with replacement of the culture medium every two days. An aliquot comprising 100 μl of Cell Counting Kit-8 solution was dripped to each well and incubated for 1 hour, followed by measuring the absorbance value at 450 nm (Elx800; BioTek Instruments, Inc., Winooski, VT, USA). For the colony formation assay, the indicated numbers of cells were plated into 6-well plates. Fourteen days later, the cells were fixed with 4% formaldehyde, stained with 1% crystal violet (Sigma-Aldrich, USA), and photographed. The Alpha Innotech imaging system (Singapore Alphatron Asia Co., Ltd.) was used to count and analyze the colonies.

### Transwell assay

The bottom membrane was coated with Matrigel from BD Biosciences for Transwell invasion assays. Transwell chamber without Matrigel was employed for Transwell migration assays. Serum-free cell resuspension was added to the apical chamber, and serum-containing medium was added to the basolateral chamber. After incubating for 24 hours, the cells were fixed with 4% paraformaldehyde for 15 minutes and stained with crystal violet for 15 minutes. The stained cells were counted using a light microscope.

### Flow cytometric analysis

Previous stable LIHC (hepatocellular liver carcinoma) cell lines were plated in 6-well plates and then which were then treated with siRNA oligo. After two days, the cells were washed twice with cold phosphate-buffered saline (PBS, HyClone), fixed with 75% ethanol, and stored at 4°C. After 24 hours, the cells were washed and collected by cold PBS. P-phycoerythrin (PE) stain was dripped into each tube and incubated at 4°C for 30 min before the cell cycle analysis. Bar graphs were the mean ± SD of three separate experiments. ^*^p < 0.05, ^**^p < 0.01.

## RESULTS

### *C12orf75* expression levels in different types of human cancer

**Figure 1:**
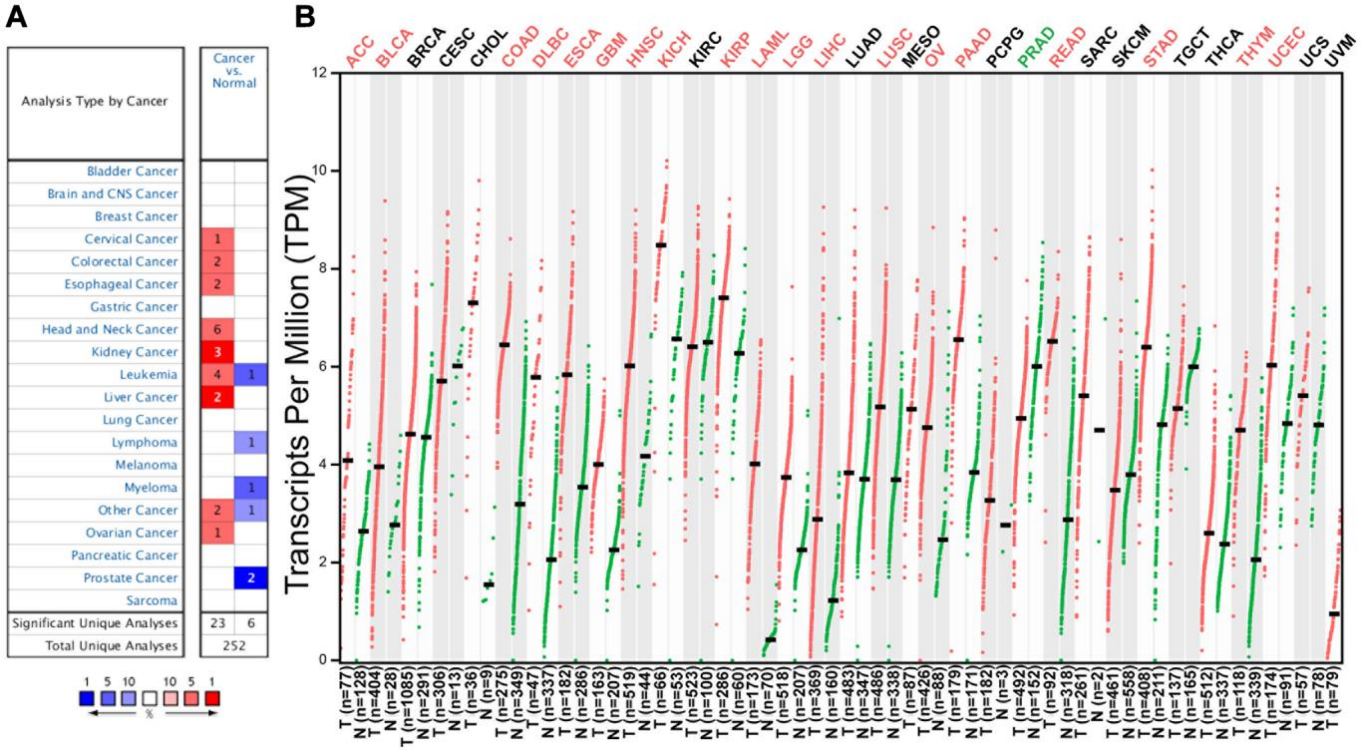
***C12orf75* expression levels in different human cancers and survival curves comparing the high and low expression of *C12orf75* in different types of cancers in the TCGA database.** (**A**) Increased or decreased expression of *C12orf75* in different cancer tissues compared with normal tissues in ONCOMINE. The number in each cell is the number of datasets. (**B**) *C12orf75* expression profile across all tumor samples and paired normal tissues determined by GEPIA. The red and green letters describe an increase and a decrease in cancer, respectively. *C12orf75*, chromosome 12 open reading frame 75; GEPIA, Gene Expression Profiling Interactive Analysis.

To assess the significance of *C12orf75* expression in cancer, the difference of *C12orf75* expression between tumor samples and matched normal tissues was analyzed using the Oncomine website. *C12orf75* was highly expressed in cervical cancer, colorectal cancer, esophageal cancer, head and neck cancer, kidney cancer, leukemia, liver cancer, and ovarian cancer. However, the expression was relatively lower in leukemia, lymphoma, myeloma, and prostate cancer (Figure 1A). The status of *C12orf75* expression in different cancers is summarized in Supplementary Table 1. The results suggested that *C12orf75* is overexpressed in most human cancers. To further estimate the variation of *C12orf75* expression among tumors and normal tissues in distinct cancer types, the GEPIA online server was utilized to investigate the RNA expression from TCGA and GTEx projects (Figure 1B). Compared to matched normal tissues, *C12orf75* was overexpressed in adrenocortical carcinoma (ACC), urothelial bladder carcinoma (BLCA), colon adenocarcinoma (COAD), diffuse large B-cell lymphoma (DLBC), esophageal carcinoma (ESCA), glioblastoma multiforme (GBM), head and neck squamous cell carcinoma (HNSC), kidney chromophobe (KICH), kidney renal papillary cell carcinoma (KIRP), acute myeloid leukemia (LAML), brain lower grade glioma (LGG), hepatocellular liver carcinoma (LIHC), lung squamous cell carcinoma (LUSC), ovarian serous cystadenocarcinoma (OV), pancreatic adenocarcinoma (PAAD), rectum adenocarcinoma (READ), stomach adenocarcinoma (STAD), thymoma (THYM), and uterine corpus endometrial carcinoma (UCEC). Meanwhile, decreased expression of *C12orf75* was in prostate adenocarcinoma (PRAD).

### Potential prognostic value of *C12orf75* in various cancers

**Figure 2:**
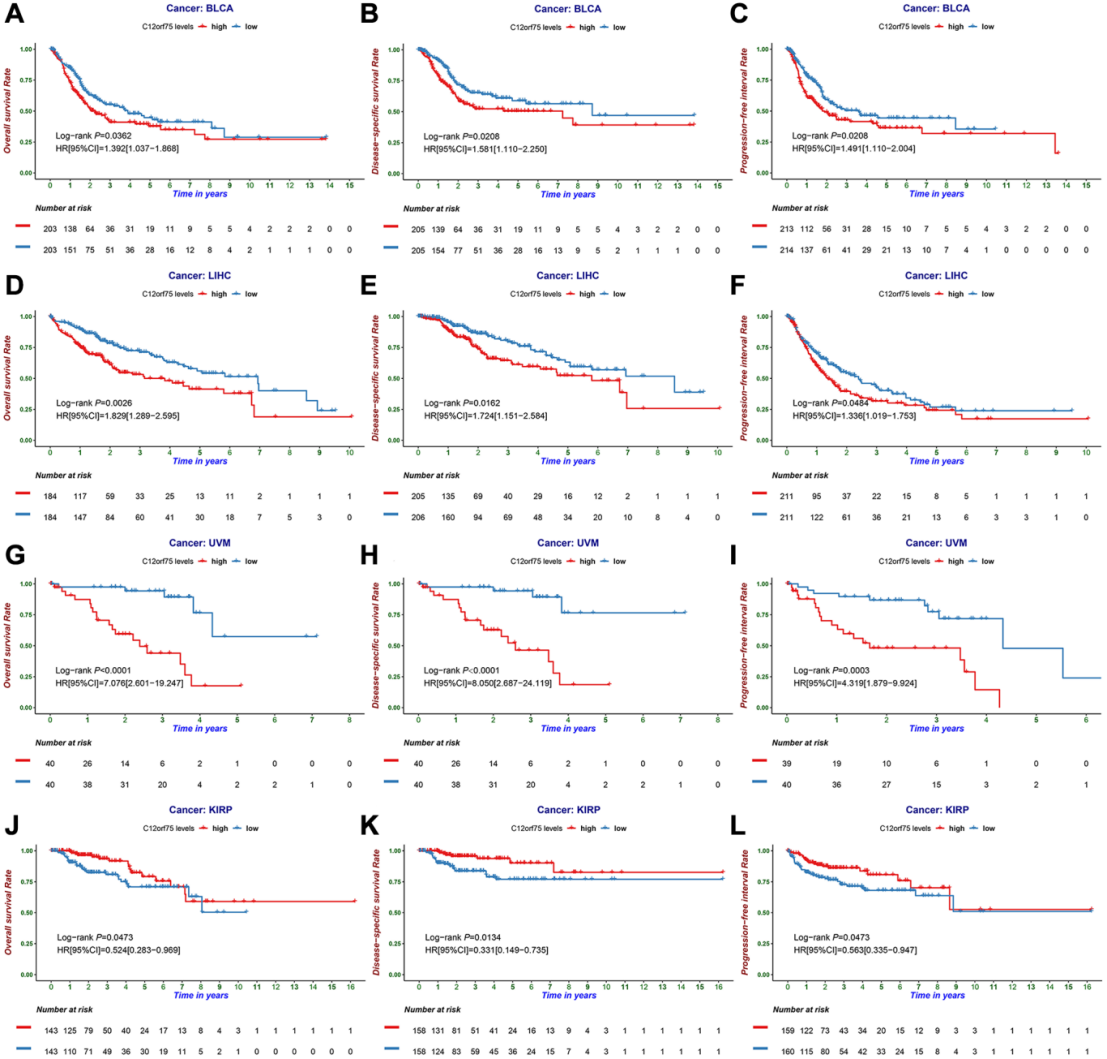
**The prognostic information of the *C12orf75* gene.** (**A**) OS, (**B**) DSS, and (**C**) PFI survival curve of bladder urothelial carcinoma (BLCA); (**D**) OS, (**E**) DSS and (**F**) PFI survival curve of liver hepatocellular carcinoma (LIHC); (**G**) OS, (**H**) DSS and (**I**) PFI survival curve of uveal melanoma (UVM); (**J**) OS, (**K**) DSS and (**L**) PFI survival curve of kidney renal papillary cell carcinoma (KIRP); OS, overall survival; DSS, disease-specific survival; PFI, progression-free interval. *P-values* from the log-rank test. *P*-values were corrected using the Benjamini-Hochberg method. *p* < 0.05 is considered as significant.

To investigate the correlation between *C12orf75* expression and patient prognosis in human cancers, we applied the *"survival"* R package to investigate the relationship between the *C12orf75* expression data of various cancers in TCGA and the patients' survival time. The results suggested that overexpression of *C12orf75* was associated with a poor prognosis in bladder urothelial carcinoma (BLCA) (OS log-rank *p* = 0.0362, hazard ratio [HR] = 1.392 [1.037–1.868]; DSS log-rank *p* = 0.0208, hazard ratio [HR] = 1.581 [1.110–2.250]; PFI log-rank *p* = 0.0208, hazard ratio [HR] = 1.491 [1.110–2.004]) (Figure 2A–2C), hepatocellular liver carcinoma (LIHC) (OS log-rank *p* = 0.0026, hazard ratio [HR] = 1.829 [1.289–2.595]; DSS log-rank *p* = 0.0162, hazard ratio [HR] = 1.724 [1.151–2.584]; PFI log-rank *p* = 0.0484, hazard ratio [HR] = 1.336 [1.019–1.753]) (Figure 2D–2F), and UVM (uveal melanoma) (OS log-rank *p* < 0.0001, hazard ratio [HR] = 7.076 [2.601–19.247]; DSS log-rank *p* < 0.0001, hazard ratio [HR] = 8.050 [2.687–24.119]; PFI log-rank *p* < 0.0003, hazard ratio [HR] = 4.319 [1.879–9.924]) (Figure 2G–2I). By contrast, the overexpression of *C12orf75* in kidney renal papillary cell carcinoma (KIRP) (OS log-rank *p* = 0.0473, hazard ratio [HR] = 0.524 [0.283–0.969]; DSS log-rank *p* = 0.0134, hazard ratio [HR] = 0.331 [0.149–0.735]; PFI log-rank *p* = 0.0473, hazard ratio [HR] = 0.563 [0.335–0.947]) (Figure 2J–2L) was associated with a better prognosis. These results indicated that high *C12orf75* expression was correlated with shorter survival time in patients with BLCA, LIHC, and UVM, but a longer survival time in KIRP. Besides, univariate and multivariate Cox survival analysis was performed to further verify the feasibility of *C12orf75* as an independent prognostic biomarker, and its expression level was still able to predict prognosis well in BLCA, KIRP, LIHC, and UVM datasets (Supplementary Tables 2–5). And, in other datasets, the hazard ratio was analyzed between the prognosis of patients with diverse cancers and the expression of *C12orf75* by PrognoScan (Supplementary Figure 1).

### Relationship between *C12orf75* expression and clinicopathological features

**Figure 3:**
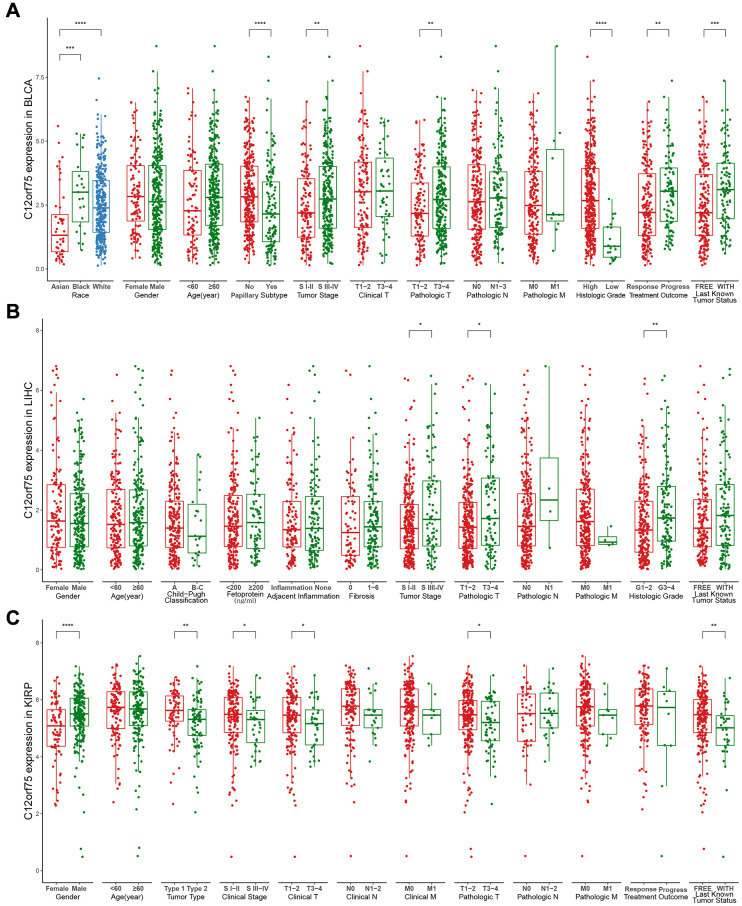
Box plot showing *C12orf75* expression levels in BLCA (**A**), LIHC (**B**) and, KIRP (**C**) of TCGA-database based on different clinicopathological features including race, gender, age, Child-Pugh classification, tumor type, papillary subtype, fetoprotein, clinical stage, tumor stage, adjacent inflammation, fibrosis, histologic grade, clinical T, clinical N, clinical M, pathologic T, pathologic N, pathologic M, treatment outcome, last known tumor status.

To better understand the influence of *C12orf75* expression on the prognosis in cancer types that showed a distinct correlation, we analyzed various clinicopathological features in selected cancers in the TCGA database using the *“ggpubr”* R package. The results suggested that in BLCA, Caucasian and Black American patients had significantly higher *C12orf75* expression than Asian patients (*p* < 0.001). The expression level of *C12orf75* was significantly higher in nonpapillary than papillary carcinoma (*p* < 0.001). Bladder cancer of tumor stage III-IV had higher *C12orf75* expression than stage I-II (*p* = 0.0089). According to the pathological staging, the expression of *C12orf75* in stage T3-4 neoplasms was dramatically higher than in stage T1-2 (*p* = 0.0035). The histological tumor grade was positively correlated with the expression of *C12orf75* (*p* < 0.001), and increased expression of *C12orf75* was positively associated with tumor progression after treatment (*p* = 0.0025). At the last follow-up, the expression of *C12orf75* was higher in patients with cancer than patients without cancer (*p* < 0.001) (Figure 3A). We next analyzed the association between clinicopathological features and RNA expression of *C12orf75* in the TCGA-LIHC datasets. Tumors in stages III-IV had noticeably higher levels of *C12orf75* than stages I-II (*p* = 0.034). The pathological stage was strongly related with the expression of *C12orf75*, with higher expression in T3-4 than T1-2 (*p* = 0.023). The RNA expression of *C12orf75* was positively correlated with the histological tumor grade in the TCGA liver cancer dataset (*p* = 0.0026) (Figure 3B). In KIRP, the expression of *C12orf75* in the tumors from female patients was significantly higher than in those from male patients (*p* < 0.001). Type 1 KIRP had an unambiguous increase of the *C12orf75* expression level in the KIRP datasets (*p* = 0.0021). However, the level of *C12orf75* transcript in KIRP tumors at an advanced clinical stage was significantly decreased compared with the early clinical stage (*p* = 0.043). From a clinicopathological viewpoint, the expression of *C12orf75* had a negative correlation with the tumor size and tumor invasion status (*p* = 0.043 and *p* = 0.034, respectively). At the last follow-up, the expression of *C12orf75* was considerably higher in patients with cancer than patients without cancer (*p* = 0.0067) (Figure 3C).

### Analysis of genetic alterations

**Figure 4:**
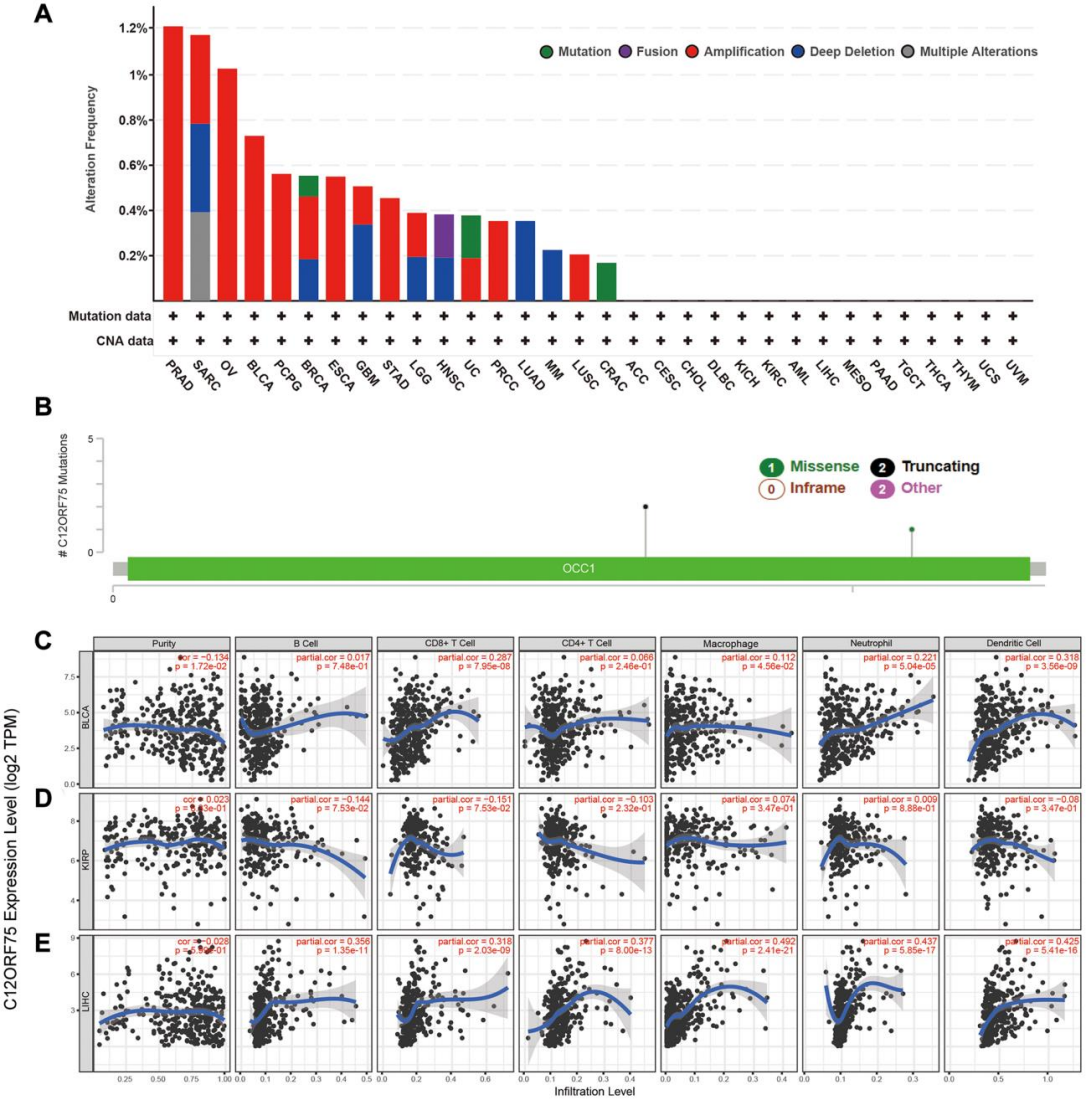
**Mutation feature of *C12orf75* in different tumors of TCGA and correlation of *C12orf75* expression with immune infiltration level in BLCA, KIRP, and LIHC.** The alteration frequency with mutation type (**A**) and mutation site (**B**) are displayed. (**C**) In BLCA, *C12orf75* expression has a significant negative correlation with tumor purity, a significant positive correlation with infiltrating levels of CD8+ T cell, macrophage, neutrophil, and dendritic cell, and no relation with infiltrating levels of B cell and CD4+ T cell. (**D**) In KIRP, *C12orf75* expression has no significant correlation with tumor purity, significant negative correlation with infiltrating levels of B cell and, CD8+ T cell, and no relation with infiltrating levels of CD4+ T cell, macrophage, neutrophil, and dendritic cell. (**E**) In LIHC, *C12orf75* expression has no significant correlation with tumor purity and, significant positive correlation with infiltrating levels of B cell, CD8+ T cell, CD4+ T cell, macrophage, neutrophil, and dendritic cell. ACC, adrenocortical carcinoma; AML, acute myeloid leukemia; BLCA, bladder urothelial carcinoma; BRCA, breast invasive carcinoma; CESC, cervical squamous cell carcinoma and endocervical adenocarcinoma; CHOL, cholangiocarcinoma; CRAC, colorectal cancer; DLBC, lymphoid neoplasm diffuse large B-cell lymphoma; ESCA, esophageal carcinoma; GBM, glioblastoma multiforme; HNSC, head and neck squamous cell carcinoma; KICH, kidney chromophobe; KIRC, kidney renal clear cell carcinoma; KIRP, kidney renal papillary cell carcinoma; LGG, brain lower grade glioma; LIHC, liver hepatocellular carcinoma; LUAD, lung adenocarcinoma; LUSC, lung squamous cell carcinoma; MESO, mesothelioma; MM, malignant melanoma; OV, ovarian serous cystadenocarcinoma; PAAD, pancreatic adenocarcinoma; PCPG, pheochromocytoma and paraganglioma; PRAD, prostate adenocarcinoma; PRCC, papillary renal cell carcinoma; SARC, Sarcoma; STAD, Stomach adenocarcinoma; TGCT, testicular germ cell tumors; THCA, thyroid carcinoma; THYM, thymoma; UC, uterine cancer; UCS, uterine carcinosarcoma; UVM, uveal melanoma. *P*-values were corrected using the Benjamini-Hochberg method. *p* < 0.05 is considered as significant.

The genetic alterations of *C12orf75* in distinct tumors among the TCGA cohorts were further studied. The highest alteration frequency of *C12orf75* (> 1.2%) was found for patients with prostate tumors and was associated with "amplification" (Figure 4A). The "amplification" type of copy number alteration (CNA) was the major type not only in prostate cancer, but also in ovarian cancer, breast cancer, bladder cancer, pheochromocytoma and paraganglioma (PCPG), esophageal cancer, stomach cancer, KIRP, and lung squamous carcinoma (Figure 4A). The types, sites, and case numbers of the *C12orf75* genetic mutations were shown in Figure 4B. Truncating mutations of *C12orf75* were the primary type of genetic alteration among them.

### Correlations between *C12orf75* expression and immune cell infiltrates

Considering the results of TCGA analysis, the status of immune infiltration in BLCA, KIRP, LIHC, MESO, UVM and UCS was further explored (Figure 4C–4E, Supplementary Figure 2). In BLCA, the *C12orf75* RNA expression had considerably positive correlations with the infiltration levels of CD8+ T cells (R = 0.287, *p* = 7.95E-08), macrophages (R = 0.112, *p* = 4.56E-02), neutrophils (R = 0.221, *p* = 5.04E-05), and dendritic cells (DCs; R = 0.318, *p* = 3.56E-9) (Figure 4C). In KIRP, the *C12orf75* expression level had no significant association with the infiltration levels of immune cells (Figure 4D). In LIHC, the *C12orf75* RNA expression had considerable positive correlations with the infiltration levels of B cells (R = 0.356, *p* = 1.35E-11), CD8+ T cells (R = 0.318, *p* = 2.03E-09), CD4+ T cells (R = 0.377, *p* = 8.00E-13), macrophages (R = 0.492, *p* = 2.41E-21), neutrophils (R = 0.437, *p* = 5.85E-17), and DCs (R = 0.425, *p* = 5.41E-16) (Figure 4E).

### Correlation between *C12orf75* expression and immune markers

The possible relationship between *C12orf75* and several immune cell markers was further explored in TIMER and GEPIA. Infiltration by distinct subtypes of T cells, such as Tfh, Th1, Th2, Th9, Th17, Th22, Treg, and exhausted T cells, was also calculated for BLCA, KIRP, LIHC, MESO, UVM, and UCS (Table 1, Supplementary Table 6). The RNA level of *C12orf75* had a consistent positive correlation with 38 of the 45 immune cell markers in BLCA, 40 of the 45 immune cell markers in LIHC, and 18 of the 45 immune cell markers in KIRP after adjusting for tumor purity. Furthermore, we explored the difference in immune markers between cancers and adjacent tissues in GEPIA. The results indicated that *C12orf75* had a consistent correlation with the infiltration levels of tumor-associated macrophages in BLCA and LIHC, while it was not significantly associated with macrophage infiltration in normal tissues. However, *C12orf75* had no substantial association with the infiltration levels of tumor-associated macrophages in KIRP (Table 2).

**Table 1:** Analysis of correlations expression between C12orf75 and Gene Markers of Immune Cells in BLCA, LIHC, and KIRP by TIMER.

**Cell type**	**Gene marker**	**BLCA (*n* = 408)**		**LIHC (*n* = 371)**		**KIRP (*n* = 290)**	
		**Cor**	***p***	**Cor**	***p***	**Cor**	***p***
B cell	CD19	0.055	**	0.209	***	−0.193	*
	MS4A1	−0.033	0.384	0.031	0.332	−0.038	0.870
	CD38	0.281	***	0.225	***	−0.169	0.091
CD8+ T Cell	CD8A	0.243	***	0.240	***	−0.148	0.214
	CD8B	0.238	***	0.226	***	−0.223	*
Tfh	CXCR5	0.020	*	0.199	***	−0.190	0.080
	ICOS	0.247	***	0.263	***	−0.083	0.612
	BCL6	−0.191	***	0.151	**	0.165	*
Th1	IL12RB2	0.419	***	0.240	***	0.337	***
	IL27RA	0.277	***	0.492	***	0.085	0.176
	TBX21	0.162	***	0.127	**	−0.089	0.635
Th2	CCR3	−0.052	0.826	0.418	***	−0.019	0.716
	STAT6	−0.159	***	0.238	***	0.489	***
	GATA3	−0.217	***	0.319	***	−0.139	*
Th9	TGFBR2	0.089	*	0.117	*	0.234	***
	IRF4	0.074	***	0.219	***	−0.152	0.111
	TNF	0.256	***	0.355	***	0.136	*
Th17	IL21R	0.225	***	0.387	***	−0.264	***
	IL23R	−0.033	0.999	0.158	**	0.131	0.111
	STAT3	0.195	***	0.272	***	0.195	**
Th22	CCR10	−0.045	0.540	0.405	***	−0.055	0.302
	AHR	−0.142	***	0.127	*	0.309	***
Treg	FOXP3	0.223	***	0.193	***	−0.129	0.108
	CCR8	0.204	***	0.392	***	−0.207	*
	IL2RA	0.272	***	0.362	***	−0.095	0.436
T cell exhaustion	PDCD1	0.171	***	0.261	***	−0.156	0.091
	CTLA4	0.192	***	0.319	***	−0.090	0.608
Macrophage	CD68	0.129	***	0.340	***	0.101	0.082
	ITGAM	0.194	***	0.586	***	−0.058	0.865
M1	NOS2	0.147	**	0.021	0.460	−0.011	0.814
	ROS1	0.229	***	0.138	0.051	−0.040	0.716
M2	ARG1	0.030	0.826	−0.237	***	0.193	*
	MRC1	0.223	***	0.032	0.566	−0.097	0.364
TAM	HLA-G	0.135	**	0.198	***	0.221	**
	CD80	0.365	***	0.397	***	−0.139	0.108
	CD86	0.243	***	0.475	***	−0.081	0.608
Monocyte	CD14	0.239	***	−0.279	***	−0.187	*
	FCGR3A	0.307	***	0.414	***	−0.132	0.149
NK	XCL1	0.154	**	0.264	***	−0.215	*
	KIR3DL1	0.068	**	0.026	0.332	−0.056	0.746
	CD7	0.164	***	0.240	***	−0.234	*
Neutrophil	FUT4	0.304	***	0.496	***	0.202	***
	MPO	0.268	***	0.090	*	−0.049	0.716
DC	CD1C	−0.090	0.831	0.168	**	0.019	0.467
	THBD	−0.088	0.822	0.170	**	0.087	0.364

Cor value of Spearman’s correlation. *p* values were corrected using the Benjamini-Hochberg method. ^*^p < 0.05; ^**^p < 0.01; ^***^p < 0.001.

**Table 2:** Analysis of correlations expression between C12orf75 and Gene Markers of B cells, macrophages, and monocytes in BLCA, LIHC, and KIRP by GEPIA.

**Cell type**	**Gene marker**	**BLCA**				**LIHC**				**KIRP**			
		**Tumor**		**Normal**		**Tumor**		**Normal**		**Tumor**		**Normal**	
		**R**	***p***	**R**	***p***	**R**	***p***	**R**	***p***	**R**	***p***	**R**	***p***
B cell	CD19	0.160	*	0.520	0.072	0.210	***	0.480	*	−0.140	0.090	0.290	0.264
	MS4A1	0.057	0.250	0.610	0.035	0.061	0.240	0.500	*	0.036	0.733	0.190	0.405
	CD38	0.360	***	0.710	*	0.240	***	0.430	*	−0.057	0.582	0.250	0.320
M1	NOS2	0.170	**	0.049	0.840	0.069	0.207	0.270	0.076	−0.058	0.456	−0.160	0.520
	ROS1	0.310	***	0.170	0.653	0.130	0.013	0.190	0.216	0.018	0.829	0.540	0.015
M2	ARG1	−0.110	0.029	0.170	0.653	−0.230	***	0.160	0.294	0.091	0.390	0.075	0.816
	MRC1	0.300	***	−0.190	0.653	0.071	0.204	0.280	0.076	0.067	0.520	−0.055	0.829
TAM	HLA-G	0.160	*	0.370	0.240	0.250	***	0.340	0.029	0.190	0.015	0.330	0.210
	CD80	0.410	***	0.550	0.056	0.380	***	0.540	**	−0.024	0.816	0.220	0.377
	CD86	0.330	***	0.430	0.163	0.440	***	0.550	**	0.041	0.733	0.330	0.210
Monocyte	CD14	0.320	***	0.053	0.840	−0.260	***	0.016	0.910	−0.098	0.390	0.040	0.830
	FCGR3A	0.370	***	−0.095	0.840	0.410	***	0.360	0.018	0.012	0.840	0.320	0.210

R value of Spearman’s correlation. *p* values were corrected using the Benjamini-Hochberg method. ^*^p < 0.01; ^**^p < 0.001; ^***^p < 0.0001.

### Analysis of the association between promoter methylation and *C12orf75* expression

Based on previous research, the association between expression of *C12orf75* and promoter methylation in different cancer types was explored using DNMIVD (Supplementary Table 7) [22, 23]. Pearson's and Spearman's correlation coefficients and the corresponding *p-*values were calculated.

### GO pathway enrichment analysis

**Figure 5:**
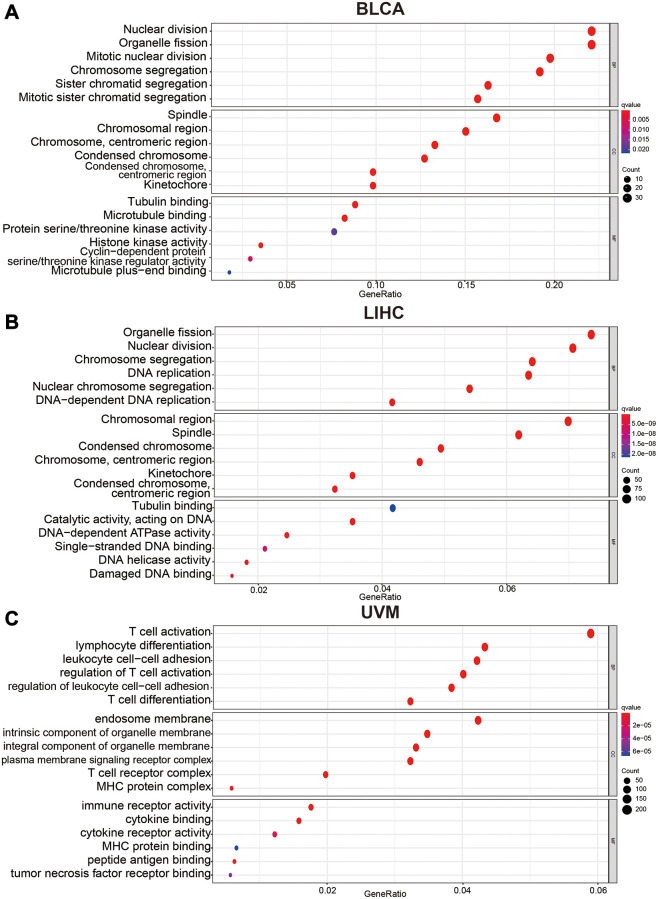
Bubble charts of Gene Ontology analysis including BP (biological process), CC (cellular component), and MF (molecular function) across *C12orf75* interactive gene lists in (**A**) BLCA, (**B**) LIHC and, (**C**) UVM. Colored by *p-*values. Bubble size represents the number of enriched genes.

To understand the influence of variety *C12orf75* expression on the biological phenotype of tumor cells, we conducted GO pathway enrichment analysis of genes. Genes associated with *C12orf75* were predicted to be involved in several biological processes, cellular components, and molecular functions. In BLCA, we found that nuclear division, organelle fission, mitotic nuclear division, chromosome segregation, sister chromatid segregation, and mitotic sister chromatid segregation were enriched in the category of biological processes, spindle, chromosomal region, centromeric region (chromosome), condensed chromosome, centromeric region (condensed chromosome) and kinetochore enriched into the category of cellular components, while tubulin binding, microtubule binding, protein serine/threonine kinase activity, histone kinase activity, cyclin-dependent protein (serine/threonine kinase activity) and microtubule plus-end binding were enriched into the category of molecular functions (Figure 5A). In LIHC, we found that organelle fission, nuclear division, chromosome segregation, DNA replication, nuclear chromosome segregation, and DNA-dependent DNA replication were enriched into the category of biological processes, chromosomal region, spindle, condensed chromosome, centromeric region (chromosome), kinetochore, centromeric region (condensed chromosome) and tubulin binding were enriched in the category of cellular components, while tubulin binding, catalytic activity (acting on DNA), DNA-dependent ATPase activity, single-stranded DNA binding, DNA helicase activity and damaged DNA binding were enriched in the category of molecular functions (Figure 5B). In UVM, we found that T cell activation, lymphocyte differentiation, leukocyte cell-cell adhesion, regulation of T cell activation, regulation of leukocyte cell−cell adhesion, and regulation of leukocyte cell−cell adhesion were enriched in the category of biological processes, endosome membrane, intrinsic component of organelle membrane, integral component of organelle membrane, plasma membrane signaling receptor complex, T cell receptor complex and, MHC protein complex were enriched in the category of cellular components, while immune receptor activity, cytokine binding, cytokine receptor activity, MHC protein binding, peptide antigen binding, and tumor necrosis factor receptor binding were enriched in the category of molecular functions (Figure 5C). These enriched pathways were positively associated with the biological characteristics of each tumor.

### Upregulation of *C12orf75* in HCC cell lines and tumor tissues

**Figure 6:**
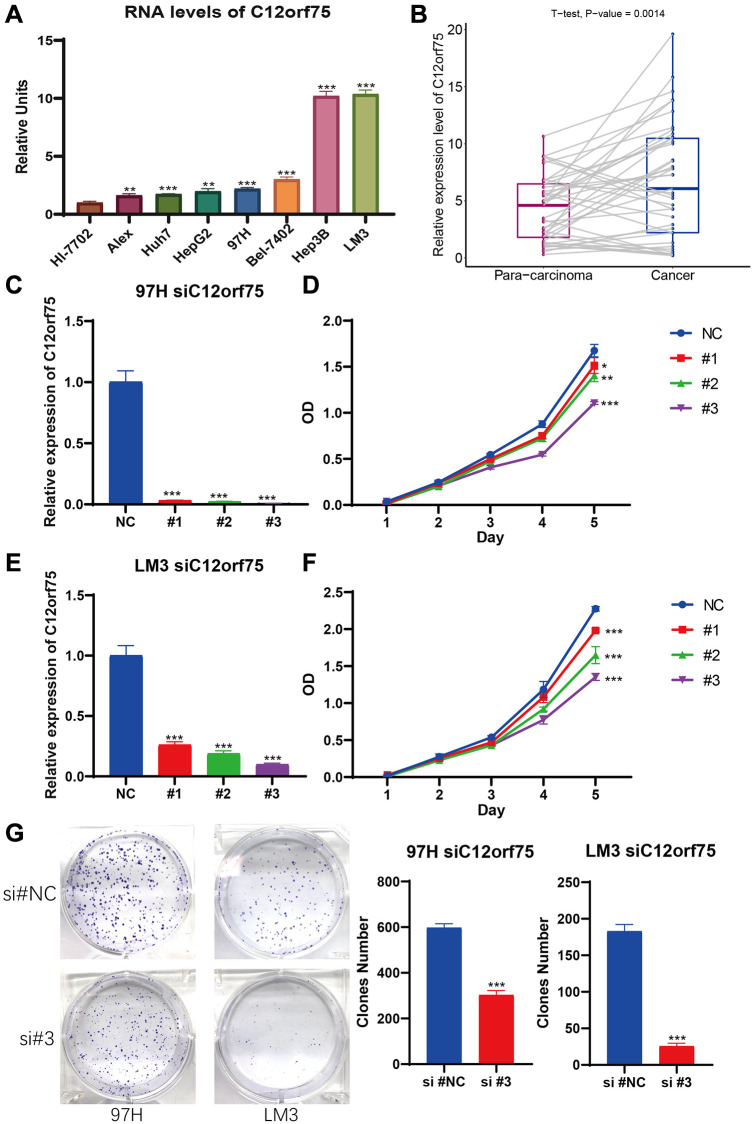
***C12orf75* was the high expression in liver cancer cell lines and patient samples and knockdown *C12orf75* suppresses LIHC proliferation.** (**A**) Analysis of *C12orf75* expression in HCC and normal liver cell lines. Compared to normal liver cell line HL-7702, *C12orf75* was remarkably upregulated in several HCC cell lines: Alex, Huh7, HepG2, 97H, Bel-7402, Hep3B, and, LM3. Fold change = log2 ∆∆Ct, log2 ∆∆Ct = (CtGAPDH − Ct *C12orf75*) of test cell lines − (CtGAPDH − Ct*C12orf75*) of Hl-7702. (**B**) Relative expression of *C12orf75* in clinical HCC and adjacent liver tissues. *C12orf75* expression was significantly higher in HCC tissues than that in adjacent liver tissues (*p* = 0.002, number of patients = 20). (**C**) RT-PCR was used to detect the efficiency of knockdown for *C12orf75* in 97H. (**D**) 97H were treated with siRNA against *C12orf75* for 24 hours and then subjected to the CCK-8 assay. (**E**) RT-PCR was used to detect the efficiency of knockdown for *C12orf75* in LM3. (**F**) LM3 were treated with siRNA against *C12orf75* for 24 hours and then subjected to the CCK-8 assay. (**G**) 97H and LM3 were treated with siRNA against *C12orf75* for 24 hours and then subjected to the colony formation assay. ^*^p < 0.05; ^**^p < 0.01; ^***^p < 0.001.

To explore the RNA expression *C12orf75* in liver cancer, RT-PCR analysis was conducted in human normal hepatocyte cell line Hl-7702 and distinct liver cancer cell lines (Alex, Huh7, HepG2, 97H, Bel-7402, Hep3B, and, LM3). As shown in Figure 6A, the expression of *C12orf75* was distinctly higher in the HCC cell lines than normal liver cell lines. Compared to Hl-7702 cells as the control, the fold change values of *C12orf75* RNA expression in HCC cells were 1.629 (Alex, *p* < 0.01), 1.746 (Huh7, *p* < 0.001), 1.978 (HepG2, *p* < 0.01), 2.208 (97H, *p* < 0.001), 3.016 (Bel-7402, *p* < 0.001), 10.206 (Hep3B, *p* < 0.001), and 10.364 (LM3, *p* < 0.001). To verify the correlation between the *C12orf75* and HCC tumorigenesis, the level of *C12orf75* expression was quantified in paired HCC lesions and corresponding adjacent normal tissues using qRT-PCR analysis. As shown in Figure 6B, the RNA expression of *C12orf75* was higher in HCC than paired adjacent normal tissues in all cases (average fold change 1.356, *p* = 0.0014). Thus, *C12orf75* might play a cancer-promoting role in the tumorigenesis and progression of HCC.

### Effect of *C12orf75* downregulation on the proliferation, migration, and invasion of HCC cells

**Figure 7:**
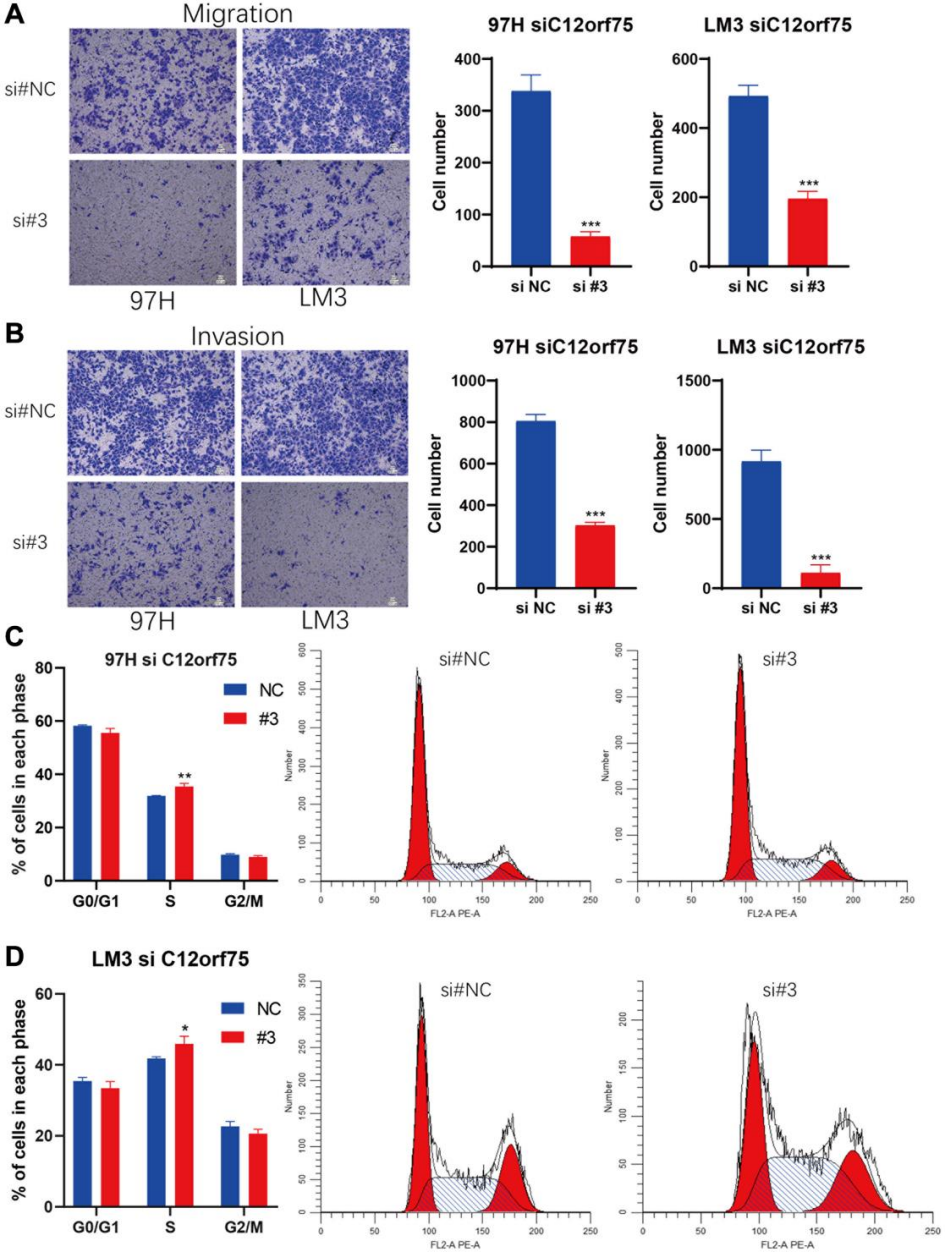
**Knockdown of *C12orf75* suppresses LIHC cell migration and invasion and arrests cell cycle.** *C12orf75* silencing suppresses cell migration (**A**) and invasion (**B**) of the 97H and LM3 (Scale bar: 200 μm.) and statistical comparisons of the indicated groups were performed. The data presented as mean ± SD from three independent experiments. Flow cytometry indicates that the knockdown of *C12orf75* suppresses S to G2 transition in 97H (**C**) and LM3 (**D**) cells. ^*^p < 0.05; ^**^p < 0.01; ^***^p < 0.001.

To mechanistically confirm the findings of expression analysis, we designed three siRNAs targeting the CDS or 3'-UTR of *C12orf75* to silence its expression. Downregulation of the RNA expression of *C12orf75* suppressed the proliferation ability of the liver cancer cells 97H and LM3 according to the CCK8 staining results (Figure 6C–6F). The third siRNA inhibited the expression of *C12orf75* to the greatest extent, and also blocked the proliferation of HCC cells most obviously. The third, strongest siRNA targeting *C12orf75* was also able to inhibit the proliferation of HCC cell lines 97H and LM3 according to the colony formation assay (Figure 6G). *C12orf75* silencing also clearly reduced the migration and invasion ability of 97H and LM3 liver cancer cells (Figure 7A, 7B).

### Knockdown of *C12orf75* leads to cell cycle arrest in HCC cells

Based on the results of RNA interference, we further explored the potential impact of *C12orf75* on the cell cycle. We performed flow cytometry on 97H and LM3 cells with *C12orf75* knockdown to examine cell cycle changes. The results showed that the S phase population was significantly increased after *C12orf75* knockdown in 97H and LM3 cells (*p* < 0.01 and *p* < 0.05, respectively). Thus, *C12orf75* knockdown induced cell cycle arrest in the S phase (Figure 7C, 7D).

## DISCUSSION

The chromosome 12 open reading frame 75 (*C12orf75*) gene was reported to be expressed and upregulated during the differentiation of mesenchymal stem cells into adipocytes. In related research in human cancers, *C12orf75* was only reported in colorectal cancer and breast cancer [24, 25]. *C12orf75* was found to inhibit CACO-2 proliferation [6], and it promoted tumor cell survival in the SW480 (Wnt+) derived from colorectal cancer. However, *C12orf75* seemed to have the opposite effect in SW480 cells (Wnt–), instead increasing apoptosis of colorectal carcinoma cells [7]. Similarly, *C12orf75* induced apoptosis in the breast cancer cell line (MCF7) [8]. These results suggest that the RNA expression of *C12orf75* is correlated with various outcomes in different cancer types and led us to explore the correlations between *C12orf75* expression and the prognosis of various human cancers.

We failed to find any other cancer-related study of *C12orf75* through a comprehensive literature search. Therefore, we conducted a comprehensive investigation of the *C12orf75* gene in distinct cancers based on TCGA and the GEO database. We found that the RNA expression of *C12orf75* was increased in most types of tumors and is obviously associated with the prognosis of patients with BLCA, KIRP, LIHC, and UVM. Overall, the upregulation of *C12orf75* expression promoted tumor progression in BLCA, LIHC, and UVM, while it seemed to be related with a better prognosis in KIRP patients (Figure 2A–2L). Thus, *C12orf75* might serve as a prognostic and diagnostic biomarker for BLCA, LIHC, MESO, UCS, and UVM.

Next, the *"ggpubr"* R package was used to study the association between different clinicopathological features and *C12orf75* expression levels according to TCGA database. Papillary and nonpapillary histological patterns of BLCA tumorigenesis were considered dual-track oncogenic pathways. The papillary type shows finger-like protrusions on the mucosal surface, and the nonpapillary tumors appear as flat lesions [26]. Even though these two patterns shared analogous chromosomal alterations, the downstream target genes vary considerably [27]. The papillary type is sometimes called papillary urothelial neoplasm because of its very low histological grade and non-invasiveness. Papillary histological types tend to have a better prognosis compared with the non-papillary type [28]. A higher level of *C12orf75* could increase the risk of worse clinicopathological stage, histological grade, and treatment outcome, which had a significant positive correlation with nonpapillary histological patterns. Similarly, a higher level of *C12orf75* expression could increase the risk of worse clinicopathological stage and histological grade in patients with HCC. These results indicated poor outcomes for BLCA and LIHC patients with relatively higher expression of *C12orf75*, and the relationship between *C12orf75* and papillary/nonpapillary track oncogenic pathways needs to be confirmed in further studies. In patients with KIRP, a higher level of *C12orf75* expression could decrease the risk of adverse clinicopathological stage and treatment outcome, which meant better outcomes for KIRP patients with relatively higher expression of *C12orf75*. These results strongly suggest that *C12orf75* could serve as a prognostic biomarker in multiple cancers.

To further explore the genomic alternations of *C12orf75* in various cancers, the genomic change data from TCGA was analyzed using cBioPortal. The results suggested that changes in the *C12orf75* gene mainly occur in prostate sarcoma and ovarian cancer, but there were only 43 cases representing less than 0.1% of patients. Thus, there was a low incidence of *C12orf75* mutations in the cancer genome [17, 18]. Further, unknown reasons for the change of *C12orf75* expression in different cancers needed further study. Additionally, we were unable to retrieve any published research that clarifies the function of *C12orf75* in tumor immunity. Understanding the tumor microenvironment may help decipher the mechanism of tumor development. As shown in Figure 4, the results of this study suggested that there is a significant correlation between tumor *C12orf75* expression and immune cell infiltration. While the expression of *C12orf75* did not show a correlation with tumor purity in KIRP and LIHC, there was a significant negative correlation with tumor purity in BLCA. Genes that are highly expressed by cells in the tumor microenvironment are considered to be negatively correlated with tumor purity. On the other hand, genes that are highly expressed in cancer cells are expected to be positively correlated with tumor purity. Our findings demonstrate that *C12orf75* expression is positively correlated with the infiltration levels of CD8+ T cells, macrophages, neutrophils, and dendritic cells (DCs) in BLCA. Similarly, *C12orf75* expression had a positive correlation with the infiltration levels of B cells, CD4+ T cells, CD8+ T cells, macrophages, neutrophils, and DCs in LIHC. Whereas *C12orf75* expression had no significant correlation with the infiltration levels of immune cells in KIRP. Thus, C12orf75 may be involved in the process of promoting immune cell infiltration in BLCA and LIHC.

In line with expectations, the expression coefficients of some immune cell markers (such as ARG1, CD14, BCL6, CCR3, and CDR10) and *C12orf75* were opposite to the expression coefficients of immune cells as a whole and *C12orf75*, which indicates that some specific immune cell subtypes may interact with *C12orf75*. In BLCA, there was a clear positive correlation between macrophage infiltration and *C12orf75* expression, and the infiltration of CD8+ T cells, which is positively correlated with *C12orf75* expression, was dominated by Th1 (T helper 1 cells) and Treg (T regulatory cells). In LIHC, both macrophages and B cells were significantly positively correlated with the expression of *C12orf75*. The infiltration of macrophages positively correlated with C12orf75 expression was dominated by TAM (Tumor-associated macrophage), in BLCA and LIHC. The recruitment of antigen-presenting cells in the tumor microenvironment is different among various cancers. Our results suggest that *C12orf75* plays an important role in immune cell infiltration and recruitment of antigen presenting cells in the tumor microenvironment, which may directly affect the prognosis and survival of patients. In the early stage of tumorigenesis, macrophages and T cells are activated to attack tumor cells and inhibit cancer progression. However, once the tumor progresses, the immune system will in turn support the tumor cells and promote cancer progression, while also inhibiting the cytotoxicity mediated by immune cells [29]. Tumor cells can induce M2-like polarization of tumor-associated macrophage (TAM) by producing lactic acid [30]. The resulting M2 macrophages can in turn promote cancer progression by releasing inflammatory mediators (including IL-6, tumor necrosis factor, interferon-γ, proteases, ROS, and nitrogen compounds) [31, 32]. Additionally, TAM can promote the migration of tumor cells via paracrine feedback, enhancing the invasion ability of tumors via cathepsins and matrix remodelling enzymes secreted by the TAM [33–35]. In BLCA and LIHC, the expression of *C12orf75* was positively correlated with a poor prognosis, which can be explained by the positive correlation between the expression of *C12orf75* and the level of TAM infiltration. As shown in Table 2, the expression of *C12orf75* in adjacent normal tissues had no significant association with immune cell infiltration, which also confirmed that *C12orf75* did change the immune cell infiltration status in the tumor microenvironment relative to adjacent normal tissues.

Further Gene Ontology enrichment analysis of biological processes, cellular components, and molecular functions revealed that the pathways closely related with the oncogenic role of *C12orf75* were enriched in BLCA and LIHC. Among them, differential genes were mainly enriched in pathways related to cell division and DNA replication.

Above all, the high expression of *C12orf75* was confirmed in liver cancer cell lines and HCC patient samples. Moreover, inhibiting the expression of *C12orf75* significantly arrested cell cycle and reduced the proliferation, migration, and invasion of HCC cells *in vitro*. According to the enrichment analysis, *C12orf75* and its positively correlated genes were enriched in DNA replication and associated pathways. DNA replication begins in the early S phase [36]. Based on these results, we further performed flow cytometry experiments on liver cancer cells with downregulated *C12orf75*. The population of liver cancer cells in the S phase was increased significantly. Liver cancer cells were blocked in the S phase by knockdown of C12orf75 and it also shows that *C12orf75* presumably involved in DNA replication or DNA damage check.

It should be noted that we only performed knockdown verification *in vitro*, but no overexpression. Moreover, only LIHC was verified. Thus, further research should verify the role of *C12orf75* in other cancers, and the levels of immune infiltration should be verified by single-cell RNA sequencing to better reflect the actual infiltration of immune cells.

## CONCLUSIONS

The differential expression of chromosome 12 open reading frame 75 (*C12orf75*) was correlated with tumorigenesis and cancer progression. The systemic analysis of *C12orf75* in the present study was performed in various types of cancer, which clarified that *C12orf75* upregulation was considerably associated with the worse survival and prognosis of patients with BLCA or LIHC. *C12orf75* may influence the cancer prognosis by changing the tumor immune infiltration and the status of tumor DNA replication. However, a positive correlation between *C12orf75* expression and the prognosis was found in KIRP patients. Finally, experiments elucidated the potential biological mechanisms of *C12orf75* in HCC. *C12orf75* has potential as a prognostic biomarker and therapeutic target for molecularly targeted drugs in urothelial bladder carcinoma, hepatocellular liver carcinoma, and renal papillary cell carcinoma.

## Supplementary Materials

Supplementary Figures

Supplementary Tables
