# Supplementary material for: Pan-cancer analysis of the prognostic value of C12orf75 based on data mining
Source: Aging (Albany NY). 2021 Jun 1;13(11):15214–39. doi: 10.18632/aging.203081 (PMC8221310; doi:10.18632/aging.203081)
Supplement: Supplementary Figures [file aging-13-203081-s001.pdf]

SUPPLEMENTARY FIGURES

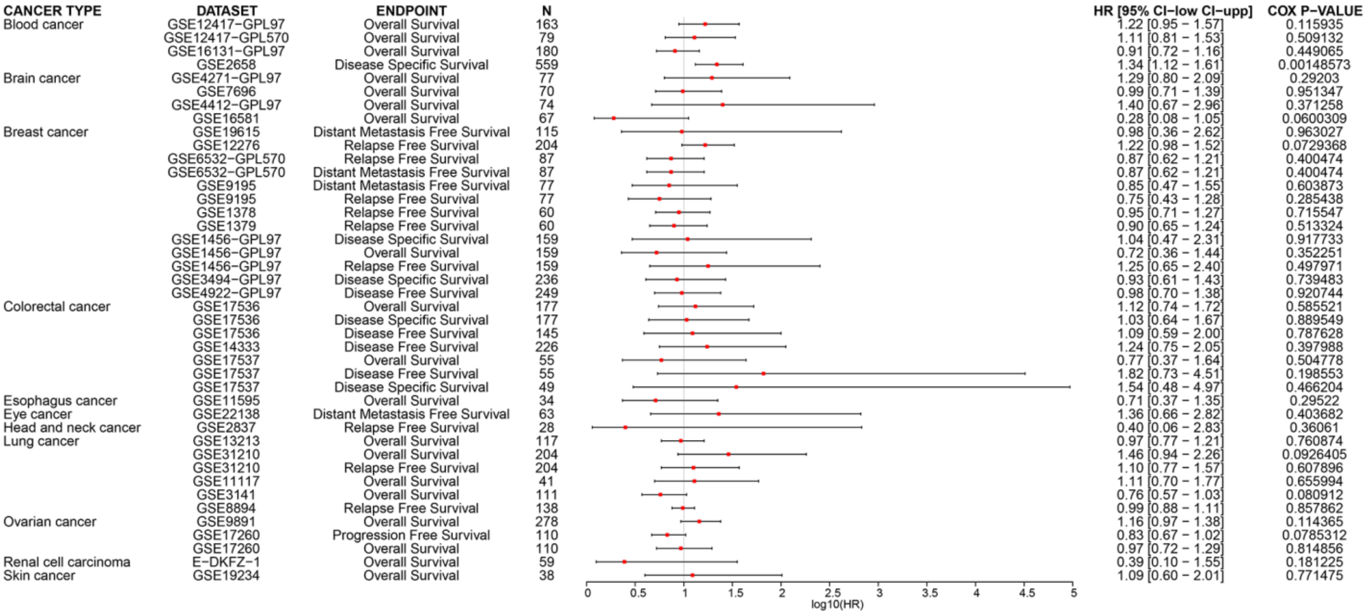

Supplementary Figure 1. The relation between *C12orf75* expression and patient prognosis of different datasets of cancers in PrognoScan. The red square represents hazard ratio (HR).

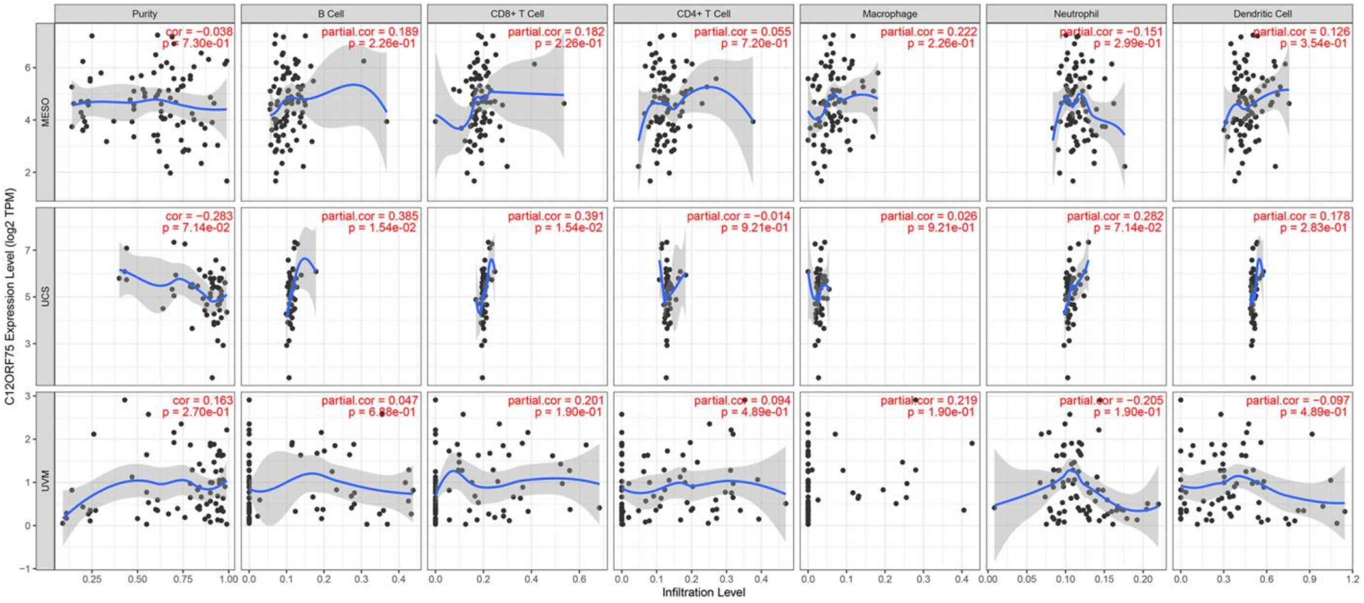

Supplementary Figure 2. Correlation of *C12orf75* expression with immune infiltration level in MESO, UCS and, UVM. *P*-values were corrected using the Benjamini-Hochberg method.
