# Supplementary material for: Pan-cancer analysis of the prognostic value of C12orf75 based on data mining
Source: Aging (Albany NY). 2021 Jun 1;13(11):15214–39. doi: 10.18632/aging.203081 (PMC8221310; doi:10.18632/aging.203081)
Supplement: Supplementary Tables [file aging-13-203081-s002.pdf]

SUPPLEMENTARY TABLES

Supplementary Table 1. *C12orf75* expression in cancerous versus normal tissue in ONCOMINE.

Cancer Site	Cancer Type	*P* Value	Sample Size	Fold Change	Up/Down Regulated Gene Rank	Reference (PMID)
Cervical	Cervical Cancer	1.22E-09	20	5.543	368 (2%)	17510386
Colorectal	Colon Carcinoma	2.36E-08	5	2.962	479 (3%)	20957034
	Colon Adenoma	8.79E-06	5	1.8	861 (5%)	20957034
Esophageal	Esophageal Squamous Cell Carcinoma	9.13E-14	51	2.423	596 (4%)	21385931
Head and Neck	Tongue Carcinoma	2.68E-07	15	4.791	199 (2%)	17510386
	Oropharyngeal Carcinoma	2.96E-05	6	5.965	361 (2%)	17510386
	Oral Cavity Carcinoma	1.83E-04	4	6.719	411 (3%)	17510386
	Floor of the Mouth Carcinoma	5.45E-04	5	7.062	1698 (9%)	17510386
	Oral Cavity Squamous Cell Carcinoma	6.59E-13	57	2.582	428 (3%)	21853135
	Thyroid Gland Papillary Carcinoma	2.69E-04	9	1.74	532 (3%)	16365291
Kidney	Chromophobe Renal Cell Carcinoma	7.02E-06	4	9.054	28 (1%)	19445733
	Renal Oncocytoma	2.66E-05	4	9.057	155 (1%)	19445733
	Papillary Renal Cell Carcinoma	7.24E-05	19	3.332	484 (3%)	19445733
Leukemia	T-Cell Acute Lymphoblastic Leukemia	2.98E-08	10	3.713	227 (3%)	17410184
	B-Cell Acute Lymphoblastic Leukemia	2.00E-12	86	2.787	283 (3%)	17410184
	T-Cell Acute Lymphoblastic Leukemia	4.23E-30	174	2.379	718 (4%)	20406941
	Pro-B Acute Lymphoblastic Leukemia	5.46E-19	70	2.343	1075 (6%)	20406941
	Chronic Myelogenous Leukemia	2.90E-13	76	–1.654	337 (2%)	20406941
Liver	Hepatocellular Carcinoma	4.88E-07	35	3.019	367 (2%)	17393520
Lymphoma	Germinal Center B-Cell-Like Diffuse Large B-Cell Lymphoma	8.54E-04	16	–1.786	251 (9%)	10676951
Myeloma	Monoclonal Gammopathy of Undetermined Significance	4.87E-07	44	–2.004	265 (2%)	17023574
Melanoma	Skin Squamous Cell Carcinoma	2.20E-04	11	3.278	917 (5%)	18442402
Seminoma	Mixed Germ Cell Tumor	1.01E-07	41	–2.284	1681 (10%)	16424014
Ovarian	Ovarian Clear Cell Adenocarcinoma	7.22E-04	7	4.867	285 (2%)	15161682
Prostate	Prostate Carcinoma	1.28E-15	62	–2.285	75 (1%)	14711987
	Prostate Carcinoma	6.78E-04	13	–3.022	547 (3%)	19737960

Supplementary Table 2. Prognostic analysis of *C12orf75* expression for overall survival in the clinicopathologic features of TCGA-BLCA.

Variable	Group A vs. Group B				Univariate Cox		Multivariate Cox	
	Group A	Total No. Patients (%)	Group B	Total No. Patients (%)	HR (95% CI)	*P*	HR (95% CI)	*p*
C12orf75	High Group	203 (50%)	Low Group	203 (50%)	1.39 (1.04–1.87)	0.027	2.28 (1.13–4.58)	0.021
Age	≥60	356 (78%)	<60	98 (22%)	1.96 (1.28–3.02)	0.002	1.59 (0.54–4.72)	0.403
Extracapsular Extension Status	Focal	38 (68%)	Extensive	18 (32%)	1.15 (0.5–2.63)	0.749		
Clinical T	T 3–4	63 (32%)	T 1-2	132 (68%)	1.44 (0.9–2.31)	0.127		
Diagnosis Subtype	Papillary	141 (31%)	On-Papillary	307 (69%)	0.69 (0.49–0.98)	0.035	1.46 (0.66–3.22)	0.349
Disease Extracapsular Extension	YES	77 (44%)	NO	97 (56%)	1.29 (0.83–2.01)	0.255		
Gleason Score	>7	32 (35%)	≤6	60 (65%)	0.89 (0.46–1.74)	0.74		
Lymphovascular Invasion	YES	170 (55%)	NO	139 (45%)	2.33 (1.6–3.39)	<0.001	2.3 (0.98–5.39)	0.056
Histologic Grade	High	429 (95%)	Low	21 (5%)	2.95 (0.73–11.93)	0.111		
Lymphonodus Positive	Positive	134 (40%)	Negative	198 (60%)	2.02 (1.46–2.81)	<0.001	1.39 (0.62–3.12)	0.42
Pathologic M	M1	11 (5%)	MO	220 (95%)	1.08 (0.73–1.6)	0.7		
Pathologic T	T3-4	288 (69%)	T1-2	132 (31%)	3.22 (1.54–6.73)	0.001	1.37 (0.38–4.93)	0.632
Gender	Male	330 (73%)	Female	124 (27%)	1.28 (0.68–2.43)	0.44		
Race	Asian	44 (11%)	White	366 (89%)	0.86 (0.62–1.19)	0.353		
Tumor Stage	Stage III-IV	311 (69%)	Stage I-II	141 (31%)	0.62 (0.31–1.21)	0.156		
BMI	≥25	223 (58%)	<25	161 (42%)	2.26 (1.56–3.27)	<0.001	0.95 (0.14–6.47)	0.961
Number Pack Years Smoked	>30	118 (47%)	≤30	132 (53%)	1.03 (0.74–1.43)	0.87		

P value for two-sided Wald test; Abbreviations: HR: hazard ratio; CI: confidence interval; BMI: Body Mass Index.

Supplementary Table 3. Prognostic analysis of *C12orf75* expression for overall survival in the clinicopathologic features of TCGA- LIHC.

Variable	Group A vs. Group B				Univariate Cox		Multivariate Cox	
	Group A	Total No. Patients (%)	Group B	Total No. Patients (%)	HR (95% CI)	*p*	HR (95% CI)	*p*
C12orf75	High Group	184 (50%)	Low Group	184 (50%)	1.83 (1.29–2.6)	0.001	1.87 (1.19–2.96)	0.007
Adjacent Tissue Inflammation Extent	YES	146 (47%)	NO	162 (53%)	1.15 (0.71–1.87)	0.564		
Age	≥60	270 (58%)	<60	198 (42%)	1.19 (0.84–1.69)	0.327		
Albumin	<3.5g/dl	87 (23%)	≥3.5g/dl	291 (77%)	1.12 (0.69–1.83)	0.651		
Child-Pugh Classification	B+C(1)	33 (11%)	A	271 (89%)	1.64 (0.81–3.33)	0.164		
Creatinine	>1.2mg/dl	57 (15%)	≤1.2mg/dl	327 (85%)	0.89 (0.5–1.6)	0.699		
Fetoprotein	≥200ng/ml	96 (27%)	<200ng/ml	259 (73%)	1.1 (0.69–1.75)	0.691		
Fibrosis Ishak Score	1~6	167 (58%)	0	119 (42%)	0.74 (0.45–1.22)	0.238		
Neoplasm Histologic Grade	G3-4	166 (36%)	G1-2	295 (64%)	1.11 (0.78–1.59)	0.557		
Pathologic M	M1	6 (2%)	M0	337 (98%)	3.96 (1.24–12.58)	0.012	3.54 (1.03–12.14)	0.044
Pathologic N	N1	6 (2%)	N0	312 (98%)	2 (0.49–8.17)	0.323		
Pathologic T	T3-4	123 (26%)	T1-2	343 (74%)	2.56 (1.8–3.64)	<0.001	1.68 (0.23–12.39)	0.611
Platelet	<150	89 (23%)	≥150	302 (77%)	0.94 (0.57–1.53)	0.797		
Family Cancer History	YES	162 (40%)	NO	248 (60%)	1.19 (0.82–1.72)	0.352		
Vascular Tumor	YES	134 (33%)	NO	270 (67%)	1.32 (0.87–2)	0.188		
Gender	Male	309 (66%)	Female	160 (34%)	0.8 (0.56–1.14)	0.219		
Race	Asian	171 (40%)	White	257 (60%)	0.76 (0.52–1.1)	0.143		
Tumor Stage	Stage III-IV	116 (27%)	Stage I-II	319 (73%)	2.46 (1.7–3.57)	<0.001	1.05 (0.14–7.77)	0.958
BMI	≥25	208 (50%)	<25	209 (50%)	0.81 (0.56–1.18)	0.28		
Weight of Samples	>160g	229 (49%)	≤160g	240 (51%)	3.21 (2.23–4.62)	<0.001	3.59 (2.19–5.87)	<0.001

P value for two-sided Wald test; Abbreviations: HR: hazard ratio; CI: confidence interval; BMI: Body Mass Index.

Supplementary Table 4. Prognostic analysis of *C12orf75* expression for overall survival in the clinicopathologic features of TCGA- UVM.

Variable	Group A vs. Group B				Univariate Cox		Multivariate Cox	
	Group A	Total No. Patients (%)	Group B	Total No. Patients (%)	HR (95% CI)	*p*	HR (95% CI)	*p*
C12orf75	High Group	40 (50%)	Low Group	40 (50%)	7.08 (2.6–19.25)	<0.001	5.88 (1.94–17.8)	0.002
Age	≥60	44 (55%)	<60	36 (45%)	1.84 (0.79–4.27)	0.151		
Clinical T	T4	38 (49%)	T2-3	40 (51%)	2.42 (0.96–6.06)	0.052		
Clinical Stage	Stage III-IV	44 (55%)	Stage II	36 (45%)	1.72 (0.7–4.19)	0.229		
Extrascleral Extension	YES	7 (9%)	NO	68 (91%)	4.64 (1.5–14.37)	0.003	2.02 (0.62–6.57)	0.242
Pathologic T	T4	34 (42%)	T2-3	46 (58%)	1.74 (0.76–3.99)	0.187		
Radiation Therapy	YES	8 (12%)	NO	58 (88%)	2.71 (0.68–10.87)	0.143		
Tumor Basal Diameter	≥17	40 (51%)	<17	39 (49%)	2.49 (1–6.18)	0.043	2.97 (0.75–11.75)	0.121
Tumor Shape	Mushroom	13 (27%)	Dome	36 (73%)	0.58 (0.16–2.06)	0.392		
Gender	Male	45 (56%)	Female	35 (44%)	1.54 (0.65–3.65)	0.321		
Tumor Stage	Stage III-IV	40 (51%)	Stage I-II	39 (49%)	1.5 (0.63–3.58)	0.356		
BMI	≥25	36 (68%)	<25	17 (32%)	1.17 (0.37–3.68)	0.789		
Weight of Samples	>40	37 (48%)	≤40	40 (52%)	1.11 (0.47–2.61)	0.812		

P value for two-sided Wald test; Abbreviations: HR: hazard ratio; CI: confidence interval; BMI: Body Mass Index.

Supplementary Table 5. Prognostic analysis of *C12orf75* expression for overall survival in the clinicopathologic features of TCGA- KIRP.

Variable	Group A vs. Group B				Univariate Cox		Multivariate Cox	
	Group A	Total No. Patients (%)	Group B	Total No. Patients (%)	HR (95% CI)	*p*	HR (95% CI)	*p*
C12orf75	High Group	143 (50%)	Low Group	143 (50%)	0.52 (0.28–0.97)	0.036	0.3 (0.11–0.82)	0.019
Age	≥60	224 (59%)	<60	154 (41%)	1.04 (0.56–1.94)	0.888		
Clinical T	T3-4	57 (21%)	T1-2	210 (79%)	4.66 (2.28–9.53)	<0.001	1.53 (0.44–5.4)	0.507
Hemoglobin	Low	128 (48%)	Normal	141 (52%)	4.4 (1.88–10.26)	<0.001	2.59 (1.04–6.46)	0.041
Lactate Dehydrogenase	Elevated	11 (23%)	Normal	36 (77%)	1.88 (0.36–9.77)	0.443		
Laterality	Right	168 (44%)	Left	210 (56%)	0.74 (0.39–1.38)	0.342		
Pathologic T	T3-4	91 (24%)	T1-2	286 (76%)	5.07 (2.76–9.3)	<0.001	2.73 (0.77–9.65)	0.118
Serum Calcium	Low + Elevated	68 (29%)	Normal	169 (71%)	1.66 (0.75–3.66)	0.205		
White Cell Count	Low + Elevated	72 (27%)	Normal	196 (73%)	1.44 (0.67–3.09)	0.353		
Gender	Male	282 (74%)	Female	99 (26%)	0.63 (0.33–1.21)	0.164		
Race	Black	75 (21%)	White	277 (79%)	0.92 (0.41–2.09)	0.853		
BMI	≥25	208 (76%)	<25	67 (24%)	0.71 (0.32–1.55)	0.39		
Pack Years Smoked	≥24	43 (49%)	<24	45 (51%)	3.38 (0.71–16.06)	0.104		
Initial Weight	>170	101 (49%)	≤170	107 (51%)	0.81 (0.36–1.83)	0.615		
Longest Dimension	>1.7	36 (21%)	≤1.7	137 (79%)	0.79 (0.26–2.37)	0.672		

P value for two-sided Wald test; Abbreviations: HR: hazard ratio; CI: confidence interval; BMI: Body Mass Index.

Supplementary Table 6. Analysis of correlations expression between *C12orf75* and Gene Markers of Immune Cells in MESO, UCS, and UVM by TIMER.

Cell type	Gene marker	MESO (*n* = 87)		UCS (*n* = 57)		UVM (*n* = 80)	
		Cor	*p*	Cor	*p*	Cor	*p*
B cell	CD19	0.136	0.353	0.102	0.354	0.233	*
	MS4A1	0.078	0.591	0.183	0.591	0.281	**
	CD38	0.140	0.353	0.339	0.354	0.419	***
CD8+ T Cell	CD8A	0.278	0.075	0.215	0.076	0.565	***
	CD8B	0.232	0.096	0.149	0.097	0.582	***
Tfh	CXCR5	0.104	0.473	–0.092	0.474	0.382	***
	ICOS	0.092	0.473	0.224	0.474	0.561	***
	BCL6	0.344	*	–0.137	*	0.252	*
Th1	IL12RB2	0.178	0.258	0.092	0.259	–0.485	***
	IL27RA	0.137	0.415	0.354	0.415	0.573	***
	TBX21	0.039	0.669	0.177	0.67	0.434	***
Th2	CCR3	0.111	0.415	0.102	0.415	0.375	***
	STAT6	–0.165	0.272	0.355	0.273	0.457	***
	GATA3	0.137	0.342	0.130	0.343	0.531	***
Th9	TGFBR2	0.265	0.080	0.185	0.08	0.243	*
	IRF4	0.071	0.591	–0.153	0.591	–0.003	0.655
	TNF	0.036	0.598	0.161	0.598	0.531	***
Th17	IL21R	0.235	0.090	–0.055	0.091	0.524	***
	IL23R	0.009	0.821	0.057	0.821	0.304	**
	STAT3	0.086	0.591	–0.041	0.591	0.599	***
Th22	CCR10	0.086	0.706	–0.295	0.706	0.311	**
	AHR	0.390	*	0.279	*	0.513	***
Treg	FOXP3	0.148	0.393	0.234	0.393	0.398	***
	CCR8	0.212	0.258	0.264	0.259	0.343	***
	IL2RA	0.107	0.591	0.166	0.591	0.408	***
T cell exhaustion	PDCD1	0.032	0.677	0.051	0.677	0.618	***
	CTLA4	0.061	0.591	0.155	0.591	0.482	***
Macrophage	CD68	0.083	0.591	0.173	0.591	0.244	*
	ITGAM	0.238	0.090	0.035	0.091	0.440	***
M1	NOS2	0.060	0.805	–0.119	0.806	0.258	**
	ROS1	0.288	0.050	–0.041	0.05	–0.178	0.187
M2	ARG1	–0.060	0.805	0.064	0.806	0.049	0.438
	MRC1	0.036	0.926	0.163	0.926	0.362	***
TAM	HLA-G	–0.134	0.591	0.425	0.591	0.404	***
	CD80	0.010	0.985	0.055	0.986	0.284	**
	CD86	0.155	0.346	0.121	0.347	0.586	***
Monocyte	CD14	0.134	0.353	0.170	0.354	0.552	***
	FCGR3A	0.168	0.298	0.066	0.298	0.532	***
NK	XCL1	0.171	0.258	0.037	0.259	0.391	***
	KIR3DL1	0.123	0.353	0.262	0.354	0.258	**
	CD7	0.146	0.320	0.139	0.321	0.599	***
Neutrophil	FUT4	0.488	***	–0.029	***	0.303	**
	MPO	–0.059	0.926	–0.129	0.926	0.016	0.991
DC	CD1C	0.035	0.926	0.047	0.926	0.214	*
	THBD	0.203	0.180	–0.145	0.181	0.390	***

Cor value of Spearman's correlation. *p* values were corrected using the Benjamini-Hochberg method. $^{*}p < 0.05$; $^{**}p < 0.01$; $^{***}p < 0.001$.

Supplementary Table 7. DNA Methylation Analysis of *C12orf75* in pan-cancer by DNMIVD.

DNMIVD					
**Disease**	**Gene Symbol**	**Pearson *r***	**Pearson *p* value**	**Spearman *r***	**Spearman *p* value**
BLCA	C12orf75	–0.181	0.000	–0.162	0.001
BRCA	C12orf75	–0.153	0.000	0.004	0.903
CESC	C12orf75	–0.035	0.535	0.011	0.847
CHOL	C12orf75	–0.072	0.640	–0.158	0.299
COAD	C12orf75	0.002	0.975	–0.032	0.566
ESCA	C12orf75	–0.207	0.007	–0.078	0.311
GBM	C12orf75	0.040	0.753	–0.013	0.917
HNSC	C12orf75	–0.006	0.883	0.071	0.104
KIRC	C12orf75	–0.049	0.363	–0.048	0.377
KIRP	C12orf75	0.132	0.023	0.151	0.009
LIHC	C12orf75	–0.119	0.016	–0.153	0.002
LUAD	C12orf75	–0.259	9.8e-9	–0.229	4.548e-7
LUSC	C12orf75	–0.151	0.003	–0.131	0.011
PAAD	C12orf75	–0.070	0.346	–0.119	0.111
PCPG	C12orf75	–0.040	0.591	–0.006	0.930
PRAD	C12orf75	0.038	0.380	0.043	0.319
READ	C12orf75	–0.125	0.212	–0.169	0.092
SARC	C12orf75	0.102	0.098	0.107	0.084
SKCM	C12orf75	–0.051	0.270	0.020	0.672
STAD	C12orf75	–0.183	0.001	–0.078	0.156
THCA	C12orf75	–0.118	0.005	–0.103	0.015
THYM	C12orf75	0.122	0.181	0.084	0.362
